# A cluster randomized trial of delivery of intermittent preventive treatment of malaria in pregnancy at the community level in Malawi

**DOI:** 10.1186/s12936-022-04216-4

**Published:** 2022-06-21

**Authors:** Beth L. Rubenstein, Jobiba Chinkhumba, Ethel Chilima, Collins Kwizombe, Ashley Malpass, Shelby Cash, Katherine Wright, Peter Troell, Humphrey Nsona, Fannie Kachale, Doreen Ali, Evans Kaunda, Sosten Lankhulani, Michael Kayange, Don P. Mathanga, John Munthali, Julie R. Gutman

**Affiliations:** 1 Malaria Branch, Division of Parasitic Diseases and Malaria, Center for Global Health, Centers for Disease Control and Prevention (CDC), 1600 Clifton Rd, Atlanta, GA 30329 USA; 2 University of Malawi College of Medicine, Malaria Alert Centre, Blantyre, Malawi; 3 Management Sciences for Health (MSH), Lilongwe, Malawi; 4 U.S. President’s Malaria Initiative, United States Agency for International Development (USAID), Lilongwe, Malawi; 5 U.S. President’s Malaria Initiative, United States Agency for International Development (USAID), Washington, D.C USA; 6 Malaria Branch, Division of Parasitic Diseases and Malaria, Center for Global Health, U.S. President’s Malaria Initiative, Centers for Disease Control and Prevention, Atlanta, GA USA; 7 Management Sciences for Health (MSH), Medford, MA USA; 8 Malaria Branch, Division of Parasitic Diseases and Malaria, Center for Global Health, U.S. President’s Malaria Initiative, Centers for Disease Control and Prevention, Lilongwe, Malawi; 9 Ministry of Health, Lilongwe, Malawi

**Keywords:** Malaria, Pregnancy, Community Health Workers, Malawi, Intermittent preventive treatment, Sulfadoxine-pyrimethamine

## Abstract

**Background:**

Malaria in pregnancy doubles the risk of low birthweight; up to 11% of all neonatal deaths in sub-Saharan Africa are associated with malaria in pregnancy. To prevent these and other adverse health consequences, the World Health Organization recommends administering intermittent preventive treatment in pregnancy (IPTp) with sulfadoxine–pyrimethamine for all pregnant women at each antenatal care (ANC) visit, starting as early as possible in the second trimester. The target is for countries to administer a minimum of three doses (IPTp3+) to at least 85% of pregnant women.

**Methods:**

A cluster randomized, controlled trial was conducted to assess the effect of delivery of IPTp by community health workers on the coverage of IPTp3 + and ANC visits in Malawi. Community delivery of IPTp was implemented within two districts in Malawi over a 21-month period, from November 2018 to July 2020. In control sites, IPTp was delivered at health facilities. Representative samples of women who delivered in the prior 12 months were surveyed at baseline (n = 370, December 2017) and endline (n = 687, August 2020). A difference in differences analysis was conducted to assess the change in coverage of IPTp and ANC over time, accounting for clustering at the health facility level.

**Results:**

Overall IPTp coverage increased over the study period. At baseline, women received a mean of 2.3 IPTp doses (range 0–5 doses) across both arms, and at endline, women received a mean of 2.8 doses (range 0–9 doses). Despite overall increases, the change in IPTp3 + coverage was not significantly different between intervention and control groups (6.9%, 95% CI: -5.9%, 19.6%). ANC4 + coverage increased significantly in the intervention group compared with the control group, with a difference-in-differences of 25.3% points (95% CI: 1.3%, 49.3%).

**Conclusions:**

In order to reduce the burden of malaria in pregnancy, new strategies are needed to improve uptake of effective interventions such as IPTp. While community health workers’ delivery of IPTp did not increase uptake in this study, they may be effective in other settings or circumstances. Further research can help identify the health systems characteristics that are conducive to community delivery of IPTp and the operational requirements for effective implementation.

*Trial registration*: ClinicalTrials.gov Identifier: NCT03376217. Registered December 6, 2017, https://clinicaltrials.gov/ct2/show/NCT03376217.

## Background

In 2019, malaria exposure occurred in an estimated 11.6 million out of 33.2 million pregnancies across sub-Saharan African (SSA) countries with moderate to high risk of malaria transmission (35% of all pregnancies in the region) [1]. Pregnant women are especially susceptible to malaria because of changes in their immune systems and the presence of a new organ, the placenta, with new targets for parasites to bind [2]. *Plasmodium falciparum* infection in pregnancy has many adverse health consequences, including maternal anaemia [3], and an increased risk of delivering a stillborn, premature, or low birthweight infant [4]. Up to 11% of neonatal deaths and 6% of all infant deaths in malaria-endemic SSA may be caused by malaria in pregnancy-associated low birthweight [3, 5, 6].

Pregnant women in SSA are, therefore, a key population for malaria prevention and control efforts, with a focus on three primary interventions: intermittent preventive treatment in pregnancy (IPTp) with sulfadoxine–pyrimethamine (SP), insecticide-treated nets, and effective case management of malarial illness and anemia [1]. The World Health Organization (WHO) recommends that pregnant women receive IPTp as early as possible starting at the beginning of the second trimester (13th week of pregnancy) and at every scheduled antenatal care (ANC) contact until delivery. Doses are typically delivered at health facilities and should be spaced at least one month apart [7]. The WHO target is for at least 85% of pregnant women in areas of moderate to high transmission of malaria to receive a minimum of three doses (IPTp3+). As of 2019, IPTp1 + coverage in sub-Saharan Africa was 62% and IPTp3 + coverage was 34% [1]. This suboptimal coverage of pregnant women with IPTp in the SSA region underscores the need for new evidence-based strategies to improve IPTp uptake.

Malawi was the first country to adopt IPTp-SP in 1993 [8], and has recommended women receive three or more doses since 2013 [9]. In 2017, 41.1% of women who recently gave birth in Malawi had received IPTp3+ [10]. This level is above average for sub-Saharan Africa, but still well below the WHO target. However, coverage with IPTp2 + in Malawi was 76.1%, suggesting the IPTp3 + target could be achieved with additional resources and innovative programmatic approaches [10]. Early initiation of ANC and ANC attendance have both been positively associated with IPTp uptake in Malawi [11].

Several studies have suggested that community health workers (CHWs) might be able to effectively deliver IPTp. A cluster randomized controlled trial in Nigeria found that training CHWs to deliver IPTp and provide ANC referrals increased the proportion of pregnant women taking IPTp2 + by 35.3% points [12]. Similar trials increased IPTp2 + uptake in Uganda by 37.3% points and IPTp3 + uptake in Burkina Faso by 17.6% points [13, 14]. The impact on ANC attendance was variable, with a statistically significant increase in number of visits in Uganda, but no effect in Nigeria or Burkina Faso [12–14]. Trials in Uganda and Malawi that focused exclusively on training CHWs for IPTp delivery without emphasizing ANC referrals also led to increased IPTp2 + uptake (27.6 and 29.3% points, respectively), but ANC attendance decreased (19.3 and 17.9% points, respectively) [15, 16]. The primary outcome in most of the previous studies was IPTp2+, rather than IPTp3+, and their generalizability and replicability are unknown.

This study assessed whether delivery of IPTp by CHWs increases IPTp3 + uptake in Malawi, while also promoting early and regular ANC attendance at health facilities.

## Methods

### Study design

A cluster randomized, controlled trial was conducted to assess the effect of community delivery of IPTp (cIPTp) by CHWs on the coverage of IPTp and ANC. Following a baseline survey in December 2017, cIPTp was implemented in intervention sites within two districts of Malawi over a 21-month period, from November 2018 to July 2020. In control sites, IPTp was delivered at health facilities via ANC clinics, per routine practice. The endline survey was carried out in August 2020.

### Study area

**Fig. 1:**
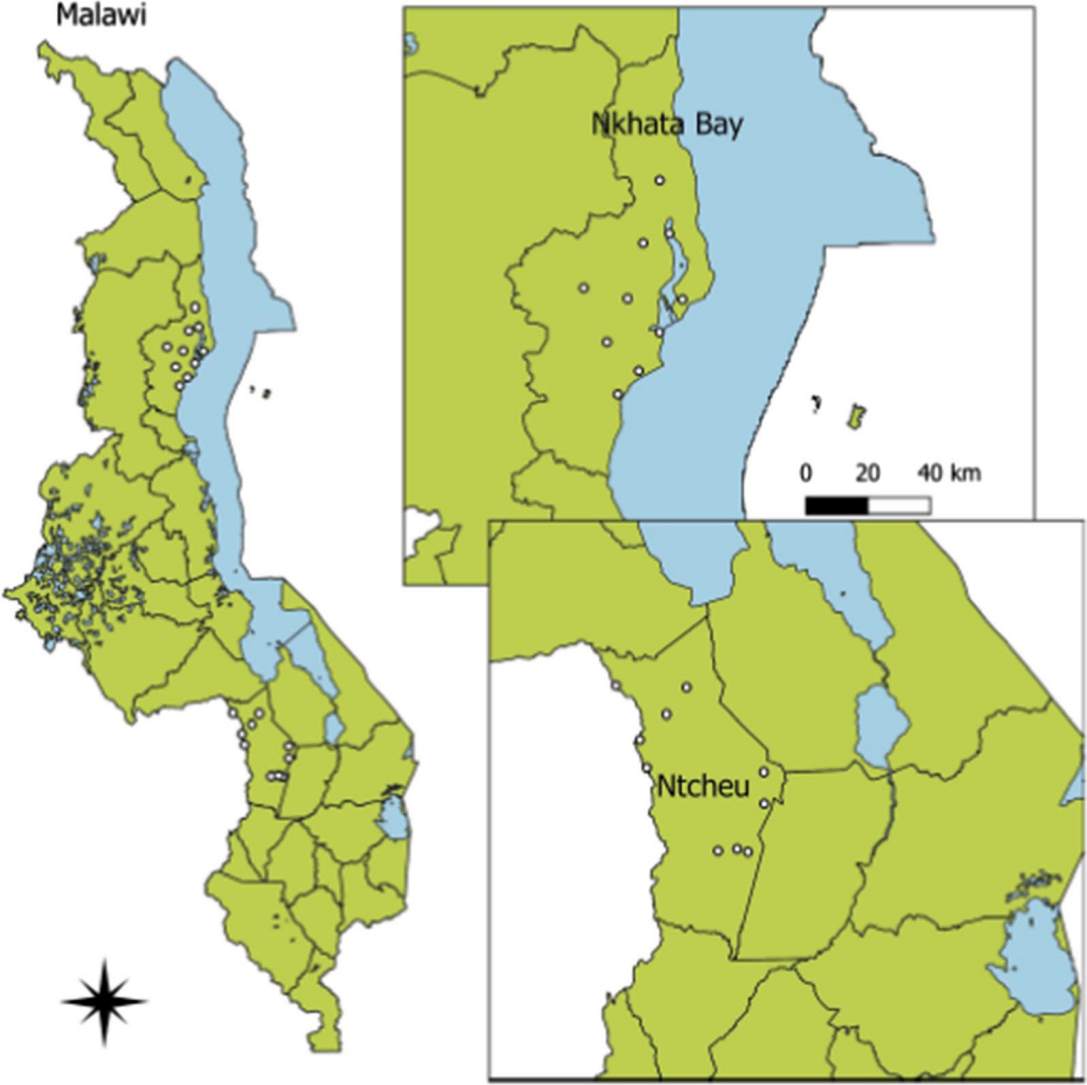
Map of the surveyed districts highlighting the location of included health facilities

The study was conducted in the districts of Ntcheu (population: 270,903), Central Region, and Nkhata Bay (population: 206,670), Northern Region, Malawi (Fig. 1). These districts were purposively selected from among the 10 districts in Malawi where the U.S. President’s Malaria Initiative (PMI) supports malaria control activities. Malaria is considered a major public health problem throughout the year in both places, with peak transmission during the rainy season from November to March. The vast majority of infections are caused by *Plasmodium falciparum* [10].

Both Ntcheu and Nkhata Bay are rural areas where most families are subsistence farmers. Nkhata Bay also has a small tourism economy. The median age of marriage for women in both districts is 18 years old, while the median age at first birth is 19 years old. Women in both districts are equally likely to deliver at a health facility (94.1% in Ntcheu and 93.6% in Nkhata Bay) and with a nurse or midwife present (76.4% in Ntcheu, 74.0% in Nkhata Bay, and for any skilled provider, 88.3% and 90.5%, respectively) [17]. Ntcheu has almost twice as many health centres and ANC providers as Nkhata Bay (40 vs. 22 and 55 vs. 29, respectively), but only about 30% more pregnant women (13,544 vs. 9905 annually) (Table 1).

**Table 1:** Characteristics of the study districts

District	Public health centres providing ANC	ANC staff	Community health workers	Total population	Pregnant population	IPTp2+ (%)^a^	IPTp3+ (%)^a^	ANC1+ (%)^a^	ANC4+ (%)^a^
Nkata Bay	22	29	87	206,670	9905	65.4	31.3	98.1	57.6
Ntcheu	40	55	81	270,903	13,544	60.5	26.6	94.2	45.9

*ANC* antenatal care, *IPTp* intermittent preventive treatment in pregnancy

^a^ National Statistical Office (NSO) and ICF Macro: Malawi demographic and health survey 2015–2016. Zomba, Malawi and Calverton, MD, USA 2017

CHWs in Malawi are the lowest cadre of service providers within the Ministry of Health. All CHWs receive a basic six-week training. Some receive further specialized trainings to provide specific services, such as integrated Community Case Management (iCCM), Community-Based Distribution of Contraceptives, Community Case Management of Acute Malnutrition, or Community-Based Maternal and Newborn Health (CBMNH). This study utilized CHWs trained in CBMNH.

Compared with Nkhata Bay, Ntcheu has fewer CHWs overall and per facility, and on average, each CHW in Ntcheu serves a larger population than the CHWs in Nkhata Bay. Although Ntcheu has fewer CHWs per facility, there are additional volunteers in the community called Secret Mothers. These volunteers receive minimal (one day) training in basic community maternal and newborn health concepts such as counselling of pregnant women on ANC attendance, use of mosquito nets, nutrition and birth preparedness, and their primary function is to encourage women to attend ANC in the first trimester.

### Intervention

In the intervention arm, all CHWs received a three-day training on how to provide IPTp to pregnant women who had already received their initial dose at the health facility, in addition to refresher training on community-based maternal and newborn health. Training covered eligibility, administration, and record keeping for the study registers and ANC cards. CHWs were encouraged to promote utilization of IPTp and ANC attendance during routine community meetings and home visits. They were also encouraged to work with community leaders, supervisors and health facility staff to identify pregnant women and organize group sessions on the importance of ANC visits and malaria prevention, including IPTp. A total of 72 CHWs were trained (49 males and 23 females). Amongst the 60 CHWs for whom data were available, the mean age was 43.4 years (standard deviation = 6.8) and had worked as CHWs for an average of 17.5 years (standard deviation = 5.0).

In addition, study personnel conducted a one-day orientation for staff at the intervention health facilities and members of the Area Development Committees to explain the objectives of the study and how CHWs would carry out their duties. Staff at the intervention health facilities were also trained to identify which pregnant women received IPTp in the community, record this in the health facility register, and report this information within the District Health Information Software (DHIS2) on a monthly basis. During the second half of implementation, the study team collaborated with local non-governmental organizations to further develop and disseminate educational messages about cIPTp.

Study personnel conducted monthly supervision at all intervention health facilities. During these visits, they met with the CHWs and reviewed their registers for completeness and accuracy, answered questions, and re-stocked SP supply. Review meetings were held in each district on approximately a quarterly basis, bringing together all CHWs to highlight successes and challenges, and collectively discuss solutions.

### Control

In the control arm, IPTp was administered exclusively at ANC by trained health facility workers, usually nurses. All CHWs received training on promotion of ANC attendance and prevention of malaria in general, in addition to refresher training on community-based maternal and neonatal health. They were not trained in cIPTp. Quarterly visits to control facilities were conducted to collect routine data on ANC attendance and IPTp administration, and to monitor SP availability.

### Selection and sample size for the cross-sectional survey

The impact of the intervention was assessed by baseline and endline cross sectional surveys, including women between the ages of 16–49 years who had a pregnancy resulting in a live birth in the previous 12 months (recently pregnant women). A three-stage cluster sampling procedure was used to select survey respondents. After excluding district hospitals, non-governmental facilities, facilities that do not provide ANC, and facilities accessible only by boat, 10 health centres were randomly selected in each district (20 in total). First, the catchment area of each health centre was designated as a cluster. Depending on geographical size, each cluster contained 3–18 enumeration areas (EAs). EAs are administrative data collection units, demarcated by the National Statistics Office, with an average of 250 households or 1,000 people. In the second stage, a single EA was randomly selected from each cluster using probability proportional to size. Finally, all households within the selected EA were listed, noting households with recently pregnant women. At baseline, simple random sampling was used to select 20 households with recently pregnant women. At endline, 40 households with recently pregnant women were randomly selected. If a selected household had more than one recently pregnant woman living in it, all women who met the inclusion criteria were included in the survey.

A sample size of 300 women (15 women per health facility catchment area) was required for each survey to achieve 80% power to detect a 30%-point difference between the group proportions for IPTp3 + coverage. These calculations assumed a baseline proportion of 30% IPTp3 + coverage (average proportion in the two districts) to an endline proportion of 60% coverage in the intervention arm. Sample size was estimated using PASS V14 (NCSS, LLC, Kaysville, UT) to assess sample size for cluster randomized trial, at a significance level of 0.05 and an intra-cluster correlation of 0.2. Sample size was then increased to allow for 30% non-response/ unavailability of the woman or ANC card which was used to assess the primary outcome measure.

### Training for cross sectional surveys

Twenty-eight enumerators took part in each of the baseline and endline cross-sectional household surveys. At baseline, all enumerators participated in a four-day training, consisting of two days of classroom instruction on ethical research conduct, consent, use of the enumeration programme, and review of the questions and sampling programme. This was followed by one day of piloting the survey in one of the non-selected EAs, and a final debriefing day, during which minor updates were made to several questions to improve the readability and clarity. At endline, an effort was made to include as many of the original data collectors as possible, and training was conducted over three days.

### Data collection and managementData collection and management

#### Cross-sectional survey

Surveys were conducted using Open Data Kit (ODK) forms hosted on the SurveyCTO platform (Dobility, Inc., Cambridge, MA). Files from SurveyCTO were exported into SAS V9.4 (SAS, Cary, NC) and STATA (STATACorp, College Station, TX) for analysis. The database was programmed with range checks and key fields were required. Data were checked daily for completeness by field supervisors before uploading to a central server. In addition, throughout the survey period, data checks for key variables were run on data downloaded from the SurveyCTO platform to ensure loops were working as expected.

#### Routine data

Visits to study and non-study health facilities were made by study supervisors monthly. During these visits, health facility and HSAs registers were reviewed and monthly data on ANC attendance, IPTp uptake, SP stocks, and other administrative data were retrospectively abstracted.

### Data analysis

A descriptive analysis was performed of the socio-demographic characteristics of recently pregnant women in the baseline and endline surveys, stratified by control and intervention areas.

The primary outcome was the change in IPTp3 + coverage over time, assessed using a difference-in-differences analysis comparing the baseline and endline cross-sectional survey data. Further difference-in-differences analyses were conducted to assess secondary outcomes, including any, two, three, or four or more ANC visits (ANC1+, ANC2, ANC3, ANC4+), IPTp1+, 2+, 4+, and 5 + coverages, number of IPTp doses, and timing of ANC initiation. Both primary and secondary outcomes were primarily based on what was recorded on the ANC card, up to a maximum of five IPTp doses. Self-reported outcomes were used only when the values from the ANC card were missing.

For binary outcomes (e.g., IPTp3+, ANC4+), difference-in-differences were calculated using a binary logistic regression model with an identity link function. Poisson regression was used for continuous outcomes (e.g., number of IPTp doses, timing of ANC initiation). In order to obtain 95% confidence intervals for the difference-in-differences, a linear model was run using the GENMOD procedure, including all the same terms as in the logistic model. All analyses accounted for clustering at the health facility level and the interaction between intervention and time. Adjusted models included gravidity, maternal age, and maternal education as additional covariates as these factors have been identified by previous studies as important determinants of ANC service use [11].

Several secondary analyses were also conducted to understand the impact of IPTp and some dynamics that could affect operationalization of the intervention. First, crude and adjusted odds of low birthweight were calculated for all women who received IPTp3+, compared with women who received fewer than three IPTp doses. The adjusted odds included gravidity, maternal age, maternal education and ANC4 + as covariates. Results were calculated for the overall sample, as well as stratified by district and survey round (baseline and endline). Second, community perceptions surrounding IPTp and ANC were summarized descriptively using several questions from the endline survey. Third, differences in IPTp3 + uptake by study arm were evaluated amongst the subset of women who lived at least five kilometres from a health facility to assess if the intervention may be more impactful in remote settings. Distance to facility was only available at endline so this analysis was based on a simple chi-square. Fourth, monthly routine service data from health facilities and visit logs from CHWs were assessed to identify potential implementation gaps.

All analyses were run in SAS Version 9.4 (SAS Institute Inc., Cary, NC) and p-values of less than 0.05 were considered statistically significant.

### Ethics

The protocol was reviewed and approved by the Malawi College of Medicine Research Ethics Committee (COMREC). Representatives from the Government of Malawi Ministry of Health were involved throughout the design and implementation of the survey, and permission was obtained from the officials in each district health office prior to initiating the survey. In each EA, permission was obtained from the village leaders. Written informed consent was obtained from each respondent before data collection. Participants were told that the survey was focused on antenatal care and malaria prevention in pregnancy. The Centers for Disease Control and Prevention (CDC) Human Subjects Office determined that CDC staff were not engaged in human subjects research for this project.

## Results

### Socio-demographic features of study participants

A total of 370 recently pregnant women were interviewed at baseline and 687 recently pregnant women were interviewed at endline. Socio-demographic characteristics of women in the control and intervention groups were similar both within and between surveys. The median age was 24 years across the sample (range 16−45 years). A third of surveyed women had one pregnancy, one quarter had two pregnancies and the remainder had three or more pregnancies. The average gestational age at first ANC visit was 20.4 weeks (standard deviation = 6.0) (Table 2).

**Table 2:** Sociodemographic characteristics of all recently pregnant survey respondents

Variable	Baseline			Endline		
	Control	Intervention	p-value	Control	Intervention	p-value
	N = 188	N = 182		N = 344	N = 343	
District			0.84			0.38
Nkhata Bay, n (%)	90 (47.9)	89 (48.9)		183 (53.2)	171 (49.9)	
Ntcheu, n (%)	98 (52.1)	93 (51.1)		161 (46.8)	172 (50.2)	
Age, in years						
Median (range)	23 (16, 44)	24 (16, 43)	0.70	24 (16, 41)	25 (16, 45)	0.14
< 20, n (%)	35 (18.6)	36 (19.8)		58 (16.9)	68 (19.8)	
20–24, n (%)	71 (37.8)	64 (35.2)		130 (37.8)	102 (29.7)	
25–29, n (%)	38 (20.2)	39 (21.4)		81 (23.6)	94 (27.4)	
30–34, n (%)	28 (14.9)	19 (10.4)		53 (15.4)	37 (10.8)	
≥ 35, n (%)	16 (8.5)	24 (13.2)		22 (6.4)	42 (12.2)	
Education			**0.03**			0.43
None, n (%)	7 (3.7)	0 (0.0)		8 (2.3)	8 (2.3)	
Primary, n (%)	135 (71.8)	131 (72.0)		243 (70.6)	257 (74.9)	
Secondary or higher, n (%)	46 (24.5)	51 (28.0)		93 (27.0)	78 (22.7)	
Gravidity*			0.39			**0.02**
1, n (%)	67 (35.8)	60 (33.0)		121 (35.2)	121 (35.3)	
2, n (%)	43 (23.0)	48 (26.4)		89 (25.9)	72 (21.0)	
3, n (%)	33 (17.7)	29 (15.9)		51 (14.8)	60 (17.5)	
4, n (%)	25 (13.4)	17 (9.3)		41 (11.9)	24 (7.0)	
5+, n (%)	19 (10.2)	28 (15.4)		42 (12.2)	66 (19.2)	
Gestation week for ANC1 visit, mean (SD)	20.2 (6.4)	20.9 (5.0)	0.32	20.3 (5.9)	20.4 (6.5)	0.88
Married, n (%)	159 (84.6)	154 (84.6)	0.99	293 (85.2)	286 (83.4)	0.52
Work outside the home, n (%)	81 (43.1)	64 (35.2)	0.12	138 (40.1)	107 (31.2)	**0.01**

* One woman was missing data on gravidity

Statistically significant p-values are bolded

### IPTp coverage

**Fig. 2:**
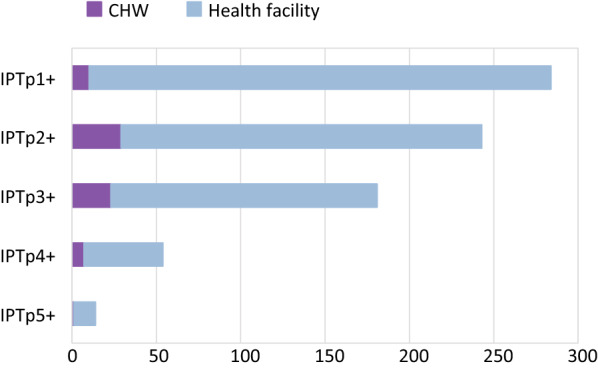
Source for intermittent preventative treatment for malaria in pregnancy (IPTp) as reported by women in the intervention arm, endline only (N = 343). *CHW* community health worker

Overall IPTp coverage increased over the study period. At baseline, women received a mean of 2.3 IPTp doses (range 0–5 doses) across both arms, and at endline, women received a mean of 2.8 doses (range 0–9 doses; only 3 women reported receiving 8 or 9 doses; this is presumed to be most likely due to error in self-reported doses). The increases in coverage occurred at all levels of dosage but were most apparent for IPTp2+ (73.8% at baseline, 83.5% at endline), IPTp3+ (50.0% at baseline, 65.6% at endline) and IPTp4+ (13.1% at baseline, 27.8% at endline). Despite overall increases, only IPTp1 + demonstrated a statistically significant increase in the intervention group compared to the control group. The difference-in-differences for IPTp1 + coverage was 13.5% points (95% CI: 4.7%, 22.3%). The change in IPTp3 + coverage did not differ significantly between intervention and control groups (6.9%, 95% CI: -5.9%, 19.6%). The change in IPTp3 + coverage was greater in Nkhata Bay (17.0%, 95% CI: -3.5%, 3.8%) compared with Ntcheu (3.5%, 95% CI: -14.9%, 21.9%), but was not statistically significant by intervention group in either district. However, the study was not powered for district-level analysis. Results did not change substantially when adjusted for gravidity, maternal age, and maternal education (Table 3). There was a statistically significant relationship between secondary education and more IPTp doses, but the other covariates were not significantly associated with IPTp outcomes (Table 4). When asked in the endline survey where they received their IPTp doses, most women reported having received IPTp from ANC rather than from the CHW (Fig. 2).

**Table 3:** IPTp and ANC coverage at baseline and endline, by study arm

	Baseline		Endline		Difference-in-differences	Crude models	Adjusted models
	Control	Intervention	Control	Intervention		p-value for DiD ^a^	p-value for DiD ^b^
	N = 188	N = 182	N = 344	N = 343			
Number of IPTp doses (mean, 95% CI)	2.3 (2.1, 2.4)	2.2 (1.9, 2.5)	2.7 (2.3, 3.1)	2.7 (2.5, 3.0)	0.2 (-0.3, 0.6)	0.51	0.39
IPTp1+ (%, 95% CI)	92.9 (89.0, 96.8)	83.0 (77.6, 88.4)	89.8 (85.8, 93.7)	93.3 (91.1, 95.6)	13.5 (4.7, 22.3)	**< 0.01**	**0.01**
IPTp2+ (%, 95% CI)	70.0 (64.1, 76.0)	71.3 (61.9, 80.8)	82.2 (76.9, 87.4)	81.0 (72.2, 89.8)	-2.5 (-16.3, 11.4)	0.73	0.86
IPTp3+ (%, 95% CI)	45.6 (38.5, 52.8)	45.1 (34.6, 55.5)	59.8 (47.0, 72.5)	66.0 (55.8, 76.3)	6.9 (-5.9, 19.6)	0.29	0.19
IPTp4+ (%, 95% CI)	16.2 (11.2, 21.1)	15.4 (7.4, 23.3)	25.8 (13.6, 38.0)	24.5 (18.8, 30.1)	-0.5 (-18.1, 17.0)	0.95	0.96
IPTp5+ (%, 95% CI)	3.1 (-0.5, 6.7)	2.8 (0.3, 5.3)	8.7 (2.9, 14.5)	6.3 (3.1, 9.4)	-2.1 (-12.1, 7.8)	0.68	0.83
Number of ANC visits (mean, 95% CI)	3.8 (3.5, 4.2)	3.4 (3.3, 3.6)	3.5 (3.1, 3.9)	3.6 (3.4, 3.9)	0.5 (-0.2, 1.3)	0.15	0.14
ANC2+ (%, 95% CI)	97.4 (93.9, 100.9)	98.4 (96.8, 100.1)	94.6 (91.2, 98.1)	95.3 (91.3, 99.4)	-0.3 (-8.4, 7.8)	0.94	0.84
ANC3+ (%, 95% CI)	92.0 (83.3, 100.8)	80.5 (75.3, 85.6)	79.9 (71.3, 88.6)	86.0 (81.3, 90.7)	17.7 (0.9, 34.4)	**0.04**	0.07
ANC4+ (%, 95% CI)	63.4 (51.8, 75.0)	46.9 (37.7, 56.1)	48.1 (36.2, 59.9)	56.8 (50.9, 62.6)	25.3 (1.3, 49.3)	**0.04**	**0.04**
Gestation week for ANC1 visit (mean, 95% CI)	18.6 (16.9, 20.4)	21.2 (20.2, 22.2)	20.3 (18.7, 22.0)	20.4 (19.6, 21.1)	-2.5 (-3.7, -1.4)	**< 0.0001**	**< 0.0001**

*IPTp* intermittent preventive treatment in pregnancy, *ANC* antenatal care visits, *DiD* difference-in-differences

Statistically significant p-values are bolded

^a^ Crude DiD models were estimated with identity link and either binomial or Poisson distribution, as appropriate

^b^ Adjusted DiD models were estimated with either with either log or logit link and binomial or Poisson distribution, as appropriate, and adjusted for gravidity, maternal age (< 20 vs. ≥ 20 years), and maternal education (secondary school or more)

**Table 4:** Effects of gravidity, maternal age, and maternal education on uptake of IPTp and ANC (N = 955)

		Regression coefficient or Odds Ratio	Confidence limits	p-value
IPTp doses	Gravidity, Primi vs. multi	0.18	(− 0.07, 0.42)	0.15
	Age < 20	0.11	(− 0.28, 0.50)	0.55
	Education, Secondary vs. less	**0.35**	**(0.13, 0.58)**	**< 0.01**
IPTp1+	Gravidity, Primi vs. multi	1.99	(0.99, 4.01)	0.05
	Age < 20	1.18	(0.57, 2.40)	0.64
	Education, Secondary vs. less	1.55	(0.92, 2.62)	0.10
IPTp2+	Gravidity, Primi vs. multi	1.08	(0.67, 1.74)	0.74
	Age < 20	1.67	(0.99, 2.83)	0.05
	Education, Secondary vs. less	**2.34**	**(1.13, 4.85)**	**0.02**
IPTp3+	Gravidity, Primi vs. multi	1.44	(0.93, 2.22)	0.09
	Age < 20	0.98	(0.55, 1.73)	0.94
	Education, Secondary vs. less	1.63	(0.96, 2.77)	0.07
IPTp4+	Gravidity, Primi vs. multi	1.03	(0.67, 1.58)	0.90
	Age < 20	0.96	(0.56, 1.63)	0.86
	Education, Secondary vs. less	1.38	(1.00, 1.91)	0.05
ANC visits	Gravidity, Primi vs. multi	0.24	(− 0.02, 0.50)	0.06
	Age < 20	0.00	(− 0.26, 0.25)	0.97
	Education, Secondary vs. less	0.16	(− 0.06, 0.38)	0.14
ANC1+	Gravidity, Primi vs. multi	0.45	(0.04, 5.43)	0.51
	Age < 20	1.20	(0.09, 15.5)	0.88
	Education, Secondary vs. less	Undefined	–	–
ANC2+	Gravidity, Primi vs. multi	0.74	(0.38, 1.42)	0.34
	Age < 20	1.43	(0.56, 3.63)	0.43
	Education, Secondary vs. less	3.82	(0.98, 14.9)	0.05
ANC3+	Gravidity, Primi vs. multi	**1.54**	**(1.02, 2.32)**	**0.04**
	Age < 20	1.05	(0.71, 1.54)	0.81
	Education, Secondary vs. less	**2.08**	**(1.37, 3.15)**	**< 0.01**
ANC4+	Gravidity, Primi vs. multi	**1.39**	**(1.05, 1.83)**	**0.02**
	Age < 20	0.86	(0.56, 1.33)	0.48
	Education, Secondary vs. less	1.14	(0.76, 1.70)	0.52
Gestation	Gravidity, Primi vs. multi	**− 1.28**	**(− 2.29, − 0.26)**	**0.02**
Week for	Age < 20	0.95	(− 0.60, 2.51)	0.21
ANC1 visit	Education, Secondary vs. less	− 0.46	(− 1.75, 0.83)	0.46

*IPTp* intermittent preventive treatment for malaria in pregnancy, *ANC* antenatal care visits, *OR* odds ratios

Statistically significant results are bolded

The crude odds of low birthweight were 59% lower for women who received IPTp3+, compared with women who received fewer than three IPTp doses (OR = 0.41, 95% CI: 0.19, 0.89). After adjusting for gravidity, maternal age, maternal education, and ANC4+, the odds of low birthweight for women who received IPTp3 + dropped to 0.47 and were no longer statistically significant (95% CI: 0.20, 1.07). The association between IPTp3 + and low birthweight in Nkhata Bay was stronger than in Ntcheu and remained statistically significant after adjusting for covariates (adjusted OR for Nkhata Bay = 0.18, 95% CI: 0.08, 0.39) (Table 5). The association between IPTp3 + and low birthweight did not differ at baseline versus endline.

**Table 5:** Effect on low birth weight of three or more doses of intermittent preventative treatment for malaria in pregnancy (IPTp3+) (N = 630)

	Overall		Nkhata Bay		Ntcheu	
	OR	Confidence limits	OR	Confidence limits	OR	Confidence limits
Crude	**0.41**	**(0.19, 0.89)**	**0.31**	**(0.14, 0.68)**	0.45	(0.15, 1.37)
Adjusted*	**0.39**	**(0.17, 0.87)**	**0.26**	**(0.11, 0.62)**	0.43	(0.13, 1.39)
Adjusted*^§^	0.47	(0.20, 1.07)	**0.18**	**(0.08, 0.39)**	0.57	(0.19, 1.78)

* Adjusted for gravidity, maternal age (< 20 vs. ≥ 20 years), and maternal education (secondary school or more)

Statistically significant results are bolded

^§^ Adjusted for four or more antenatal care visits (ANC4+)

**Table 6:** Community perceptions of intermittent preventative treatment for malaria in pregnancy and health workers, endline only (N = 687)

	Agree n (%)
The medicine given to pregnant women to prevent malaria works well to keep the mother healthy.	644 (93.7%)
I am able to take the medicine to prevent malaria at least three times during pregnancy.	653 (95.1%)
More than half of the women in my community take medicine to prevent malaria when they are pregnant.	556 (80.9%)
I find the community health workers in my community helpful.	387 (57.9%)
Health workers in the health facility in this community are knowledgeable about how to administer IPTp for pregnant women.	646 (93.3%)
Community health workers in this community treat their patients with respect.	479 (69.7%)
Heath workers that care for pregnant women with malaria in the health facility in this community treat their patients with respect.	602 (86.9%)

### ANC coverage

Overall ANC1 + and ANC2 + coverage were close to 100% at baseline and endline and ANC3 + coverage was 80.9% at baseline and 84.5% at endline. Coverage for subsequent visits declined sharply, with ANC4 + at 49.5% at baseline and 55.5% at endline and ANC5 + at 13.7% at baseline and 24.2% at endline. There was a statistically significant increase in ANC4 + coverage in the intervention group compared with the control group, with a difference-in-differences of 25.3% points (95% CI: 1.3%, 49.3%). Women in the intervention group also started ANC an average of 2.5 weeks earlier in their pregnancy, compared with women in the control group (95% CI: -3.7, -1.4). Again, adjusting for gravidity, maternal age, and maternal education did not substantially change the results (Table 3). Women who were primigravida had higher odds of completing ANC3 + and ANC4 + and started ANC an average of 1.3 weeks earlier, compared with women who were multigravida (Table 4). When asked in the endline survey where they received their IPTp doses, most women reported having received IPTp from ANC rather than from the CHW (Fig. 2).

### Community perceptions IPTp

Attitudes surrounding self-efficacy and social norms about IPTp were very favorable. Over 90% of women perceived the medicine they were given to prevent malaria works well and that they would be able to take the medicine at least three times during their pregnancy. More than 80% of women were aware that most pregnant women in their community take medicine to prevent malaria. However, when respondents were asked about the quality of interactions with community health workers, community health workers were rated as substantially less helpful and less respectful than workers at the health facility (Table 6).

### Distance to health facility

Amongst the subset of women who lived at least five kilometres from a health facility (N = 175), 67.7% of women in the intervention arm received IPTp3+ (N = 42/62), compared with 48.7% of women in the control (N = 55/113). This difference was statistically significant (p-value for chi-square = 0.02).

### Routine service data

**Fig. 3:**
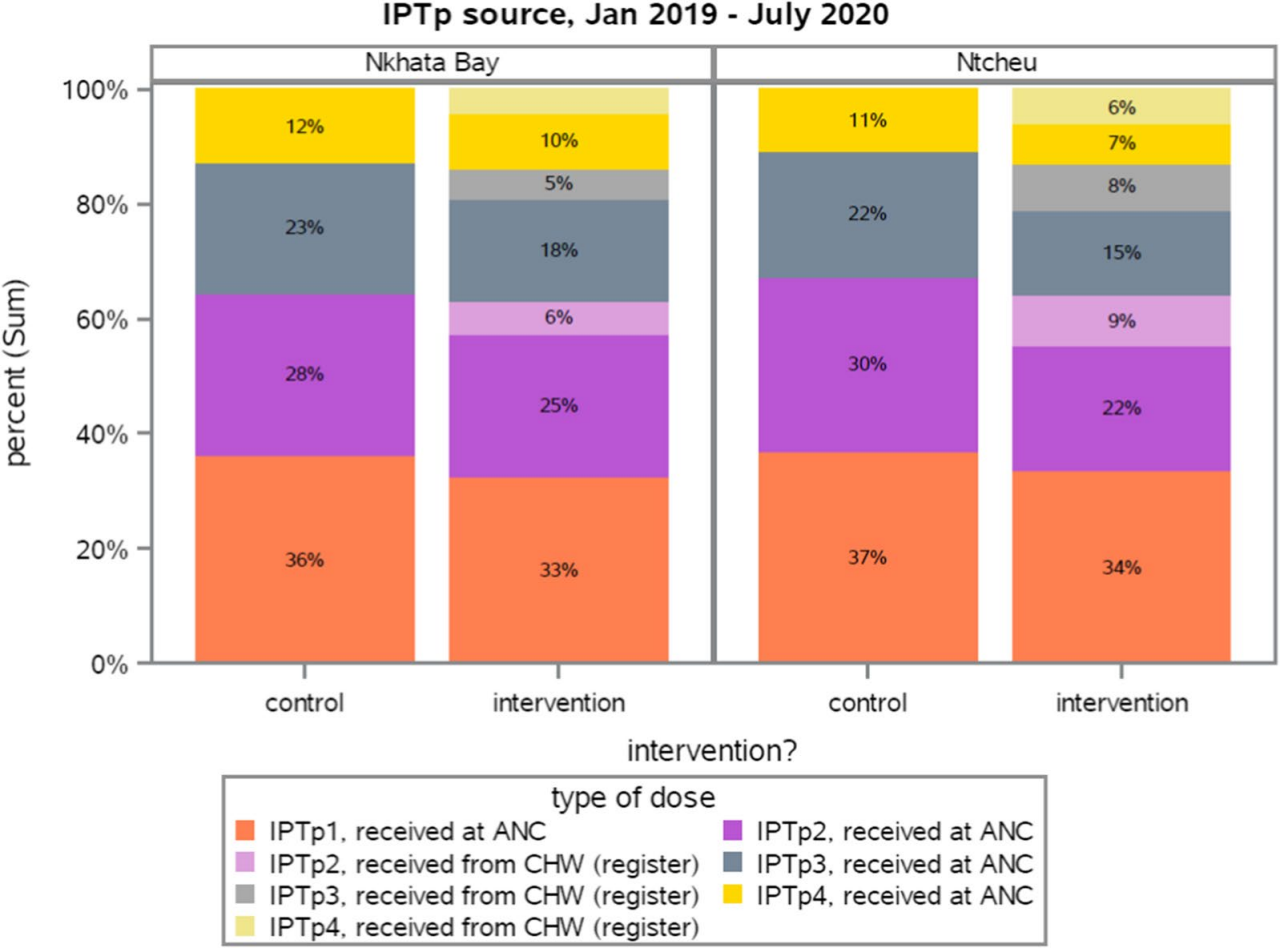
Source for intermittent preventative treatment for malaria in pregnancy (IPTp), January 2019 to July 2020, routine service data. *ANC* antenatal care, *CHW* community health worker

**Fig. 4:**
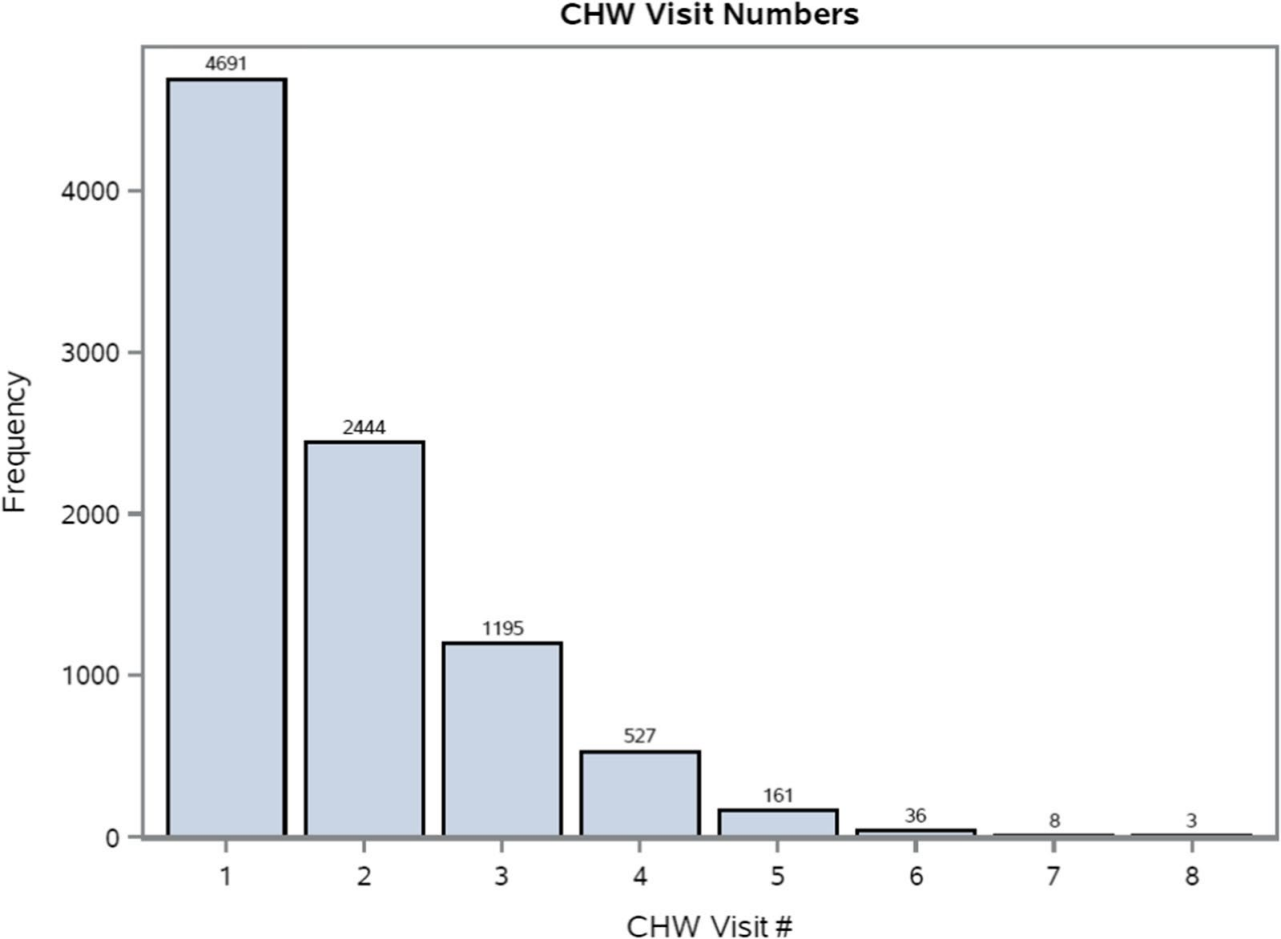
Frequency of community health worker (CHW) visits with pregnant women by visit number, January 2019 to July 2020

The routine service data showed that IPTp delivery primarily occurred at ANC in both control and intervention sites (Fig. 3). In addition, most CHWs spent most of their time conducting first visits with pregnant women, with first visits accounting for 51.8% of total visits. The frequency of second visits was roughly half the frequency of first visits, third visits were roughly half as frequent as second visits, and so on (Fig. 4). Each active CHW made an average of 6.9 visits per month, with a range of 1 to 39 visits. Both control and intervention facilities were well stocked with SP throughout the study, and study staff replenished CHWs stocks during monthly supervision, ensuring that stock-outs did not occur.

## Discussion

In a representative sample of recently pregnant women in two districts in Malawi, engaging CHWs in IPTp delivery did not result in increased IPTp3 + uptake. While the intervention did increase IPTp1 + uptake, there were no effects on other levels of dosage, including IPTp2+, IPTp4+, and overall number of doses. The intervention had a more promising effect on ANC attendance, including an increase in ANC4 + and earlier ANC initiation, compared with the control group. In contrast to cIPTp, CHWs were not providing ANC services directly, but this finding suggests that CHWs may still have a positive influence on women’s healthcare-seeking behaviour. Moreover, the finding assuages concerns that cIPTp would negatively impact ANC attendance if pregnant women perceived CHWs as a substitute for ANC. Completion of ANC4 + and early ANC initiation are strongly associated with improved outcomes for pregnant women and babies [18–20].

The null impact of the intervention on IPTp uptake is partially explained by increases in IPTp3 + coverage in the control area, likely as a result of improved SP stocks, unrelated to CHWs. During the study period, IPTp3 + coverage in the control areas increased from 45.6 to 59.8% and assuming a similar underlying trend in the intervention areas, this higher level of coverage may have meant diminishing returns for CHW outreach activities. It is possible that CHWs are more impactful in settings with lower and more stable levels of IPTp3 + coverage, such as Burkina Faso [14]. Spillover is another factor that may have biased the results toward the null if some women in the control arm received IPTp from CHWs in the intervention sites. However, in the survey, only two women in the control arm reported receiving IPTp from CHWs, suggesting spillover was very limited.

Implementation challenges were another factor that likely detracted from the intervention’s impact. Routine service data showed that very few women in the intervention sites received IPTp from CHWs. Moreover, CHWs only visited with the majority of women they assisted once. The lack of follow-up visits impeded CHWs’ ability to affect the primary outcome, IPTp3+, which requires at least two visits. This may be partially driven by an insufficient number of CHWs. Malawi recommends a ratio of 1 CHW per 1000 people [21]. The actual ratios in the study areas were 1 CHW: 1150 people in Nkhata Bay and 1 CHW: 1945 people in Ntcheu. The slightly greater increase in IPTp3 + coverage in Nkhata Bay compared with Ntcheu supports this theory, though CHW density is just one of many differences between the two districts. The COVID-19 pandemic may have compromised implementation during the final five months of the study period (March to July 2020). However, others have argued that CHWs can play a pivotal role in sustaining essential health services during a pandemic when health clinics are overwhelmed and/or inaccessible [22, 23]. Women also had less favourable perceptions of the quality of care provided by CHWs, compared with care at health facilities. These perceptions may have dissuaded women from consulting CHWs for IPTp. Finally, gender dynamics may have negatively affected IPTp uptake in the communities; nearly 70% of the CHWs in the intervention arm were male, which may have led to reduced uptake if women felt uncomfortable visiting male CHWs while pregnant.

Notably, IPTp3 + had a sizeable effect on preventing low birthweight in the study sample, even though the adjusted odds ratio was not statistically significant. Moreover, the fact that the association between IPTp3 + and low birthweight did not differ at baseline versus endline suggests that the effectiveness of IPTp has not been impeded by recent growth in SP resistance in Malawi [24]. In addition, the analysis of IPTp3 + uptake amongst women who lived at least five kilometres from a health facility suggests that CHWs may be more impactful in remote settings. However, this analysis was limited by the fact that distance to facility was only available at endline and was not balanced between study arms.

This was the first study to rigorously evaluate the effectiveness of engaging CHWs in IPTp delivery in Malawi. The study had at least four limitations. First, and likely most importantly, there were deficiencies in the operationalization of the intervention, including insufficient follow-up from CHWs. Second, although the demographics of the women in the intervention and control groups were similar, there were imbalances in baseline levels of IPTp1+, ANC3+, ANC4+, and gestation week for ANC initiation across study arms. Third, for some of the difference-in-differences analyses (especially those with study arm imbalances at baseline), the assumption that the intervention and control groups have parallel trends in outcome may not have been met. This would lead to biased estimation of the causal effect. Fourth, both IPTp doses and ANC visits were measured primarily based on ANC cards and these records may be incomplete. Assuming ANC cards had similar levels of missing data across both study arms, this would bias the results towards the null.

## Conclusions

The evidence for the effectiveness of IPTp3 + in preventing malaria in pregnancy is robust. In order to reduce the burden of malaria in pregnancy and ultimately eradicate malaria, new strategies are needed to improve uptake of this proven, effective intervention. While CHWs did not increase IPTp uptake in this study, it is possible that CHWs may be effective in increasing IPTp uptake in other settings or circumstances. In addition, CHWs likely still contribute to healthy pregnancies even when they do not increase IPTp uptake, as evidenced by the improvements in ANC attendance and early initiation in the intervention group. Further research can help identify the health systems characteristics that are conducive to CHW engagement approaches to improve IPTp delivery, such as low baseline levels of IPTp coverage, long distances to health facilities, and the optimal ratio of CHWs to population. CHW training and supervision should also emphasize the importance of follow-up visits.

## Data Availability

The datasets generated and analysed during the current study are available from the corresponding author on reasonable request.

## Abbreviations

ANC: Antenatal care
CBMNH: Community-Based Maternal and Newborn Health
CDC: Centers for Disease Control and Prevention
CHW: Community health worker
COMREC: Malawi College of Medicine Research Ethics Committee
DHIS2: District Health Information Software
EA: Enumeration area
iCCM: Integrated Community Case Management
IPTp: Intermittent preventive treatment in pregnancy
PMI: U.S. President’s Malaria Initiative
SP: Sulfadoxine–pyrimethamine
SSA: Sub-Saharan Africa
WHO: World Health Organization
